# Effects of age, body height, body weight, body mass index and handgrip strength on the trajectory of the plantar pressure stance-phase curve of the gait cycle

**DOI:** 10.3389/fbioe.2023.1110099

**Published:** 2023-02-15

**Authors:** Christian Wolff, Patrick Steinheimer, Elke Warmerdam, Tim Dahmen, Philipp Slusallek, Christian Schlinkmann, Fei Chen, Marcel Orth, Tim Pohlemann, Bergita Ganse

**Affiliations:** ^1^ German Research Center for Artificial Intelligence (DFKI), Saarbrücken, Germany; ^2^ Department of Trauma, Hand and Reconstructive Surgery, Saarland University, Homburg, Germany; ^3^ Werner Siemens-Endowed Chair for Innovative Implant Development (Fracture Healing), Saarland University, Homburg, Germany

**Keywords:** gait, motion analysis, ground reaction (forces), ageing, obesity, insoles, handgrip strengh, smart healthcare

## Abstract

The analysis of gait patterns and plantar pressure distributions *via* insoles is increasingly used to monitor patients and treatment progress, such as recovery after surgeries. Despite the popularity of pedography, also known as baropodography, characteristic effects of anthropometric and other individual parameters on the trajectory of the stance phase curve of the gait cycle have not been previously reported. We hypothesized characteristic changes of age, body height, body weight, body mass index and handgrip strength on the plantar pressure curve trajectory during gait in healthy participants. Thirty-seven healthy women and men with an average age of 43.65 ± 17.59 years were fitted with Moticon OpenGO insoles equipped with 16 pressure sensors each. Data were recorded at a frequency of 100 Hz during walking at 4 km/h on a level treadmill for 1 minute. Data were processed *via* a custom-made step detection algorithm. The loading and unloading slopes as well as force extrema-based parameters were computed and characteristic correlations with the targeted parameters were identified *via* multiple linear regression analysis. Age showed a negative correlation with the mean loading slope. Body height correlated with Fmean_load_ and the loading slope. Body weight and the body mass index correlated with all analyzed parameters, except the loading slope. In addition, handgrip strength correlated with changes in the second half of the stance phase and did not affect the first half, which is likely due to stronger kick-off. However, only up to 46% of the variability can be explained by age, body weight, height, body mass index and hand grip strength. Thus, further factors must affect the trajectory of the gait cycle curve that were not considered in the present analysis. In conclusion, all analyzed measures affect the trajectory of the stance phase curve. When analyzing insole data, it might be useful to correct for the factors that were identified by using the regression coefficients presented in this paper.

## 1 Introduction

The analysis of gait patterns and pressure distributions under the feet *via* insoles is increasingly used to study specific questions in the everyday lives of people, i.e., to analyze recovery after surgeries (Braun et al., 2017) and to monitor training or health (Subramaniam et al., 2022). Instrumented insoles have become more usable in recent years, as several technical issues could be resolved. These include durability, usability, calibration, hysteresis and drift, limited battery life and data storage capacity, and related to that, the restriction to low sample frequencies that are associated with higher error rates (North et al., 2012; Elstub et al., 2022; Subramaniam et al., 2022). Currently, the usability is still limited by the complexities of data analysis and the need for advanced algorithms and tools to be able to draw meaningful conclusions from these data (Anderson et al., 2022; Chatzaki et al., 2022). For machine-learning-based analyses, it is important to find parameters that are known to have an effect on the stance phase curve and could serve as input variables. This is why the authors conducted the present study in the first place. In addition, one could correct for such confounders when analyzing insole data. Characteristic effects of anthropometric and other individual parameters on the trajectory of the stance phase curve measured by insoles have not been previously reported.

Known gait alterations typical for higher age include a more cautious gait, reductions of the preferred walking speed, cadence, step and stride length plus width, as well as increases in speed-normalized cadence and gait speed variability (Herssens et al., 2018; Niederer et al., 2021). The underlying causes include physical performance declines in advancing age that are associated with progressive losses of muscle mass, decreased joint flexibility, declines in force- and power-generating capacity, as well as age-related changes of the cardiovascular system (Ganse and Degens, 2021). In obesity, the spatiotemporal gait parameters, parameters of pedography and joint kinematics differed compared with normal-weight matched control groups (Choi et al., 2021; Pau et al., 2021). In detail, during walking significantly greater peak pressure values were reported for the front and rear of the foot in normal compared to obese people (Choi et al., 2021). In addition, obese people had a lower gait speed and stride length, shorter stance and swing phases, as well as longer double support phases (Pau et al., 2021). The ankle, knee and hip ranges of motion were smaller in the obese compared to the non-obese (Pau et al., 2021). Compared to obese people, people who are both, obese and old walked with higher center of pressure (CoP) velocity, shorter stride, and spent more time in the support phase (Maktouf et al., 2020). Known gait changes associated with body height include negative correlations of height with cadence (every 10 cm increase in height decreased cadence by 5.6 steps/min), ankle velocity, stride time and stride length (every 10 cm increase in height extended the stride length by 5 cm) (Mikos et al., 2018). In a study with 120 healthy subjects by Senden et al. (2012), together, height, age and gender explained 51% of the variability in step length, 41% of the variability in cadence, and 34% of the variability in age. In addition, age and gender accounted for 34% of the variability in walking speed.

Muscle power is likely to influence the stance phase curve, i.e., *via* more forceful movements and a stronger or weaker push-off (Kim et al., 2022). Maximal voluntary contraction measurements of the legs due to risks of re-injury and worsening are problematic in patients with injuries or degenerative conditions of the lower extremities. In clinical settings, handgrip strength measurements are already widely established, also since they are much easier to perform, and far less time-consuming than leg force measurements. Handgrip strength varies substantially with sex, age and body height, and it correlates with the remaining years of life (Scherbov et al., 2022). Low handgrip strength and gait speed are associated with cardiovascular mortality and markers of neurodegeneration (Chainani et al., 2016; Jacob et al., 2022). It is known that stride length at preferred walking speed correlates positively with muscle mass, and the variance in the double support phase correlates with muscle strength (Kim et al., 2022).

Apart from these known characteristic gait changes, it is currently unknown how these factors affect the trajectory of the stance-phase curve derived from plantar-pressure data. The M-shaped curve of ground reaction forces during the stance phase is defined by two maxima, one minimum, the loading and unloading slope, as well as the force during defined periods (Larsen et al., 2008). We hypothesized characteristic changes of age, body height, body weight, body mass index (BMI) and handgrip strength on the plantar pressure stance-phase curve trajectory in healthy participants. In detail, we hypothesized higher forces and steeper loading and unloading slopes in younger, stronger and taller people, and in those with a higher body mass and body mass index.

## 2 Materials and methods

Ethical approval was obtained from the IRB of Saarland Medical Board (Ärztekammer des Saarlandes, Germany, application number 30/21). The study is part of the project Smart Implants 2.0 – Weight-bearing and Gait Observation for Early Monitoring of Fracture Healing and Individualized Therapy after Trauma, funded by the Werner Siemens Foundation. It is registered in the German Clinical Trials Register (DRKS-ID: DRKS00025108).

### 2.1 Measurement protocol

Data were collected from healthy volunteers. The inclusion criteria were the ability to walk on a treadmill, and age 18 years and older. Exclusion criteria were immobility, previous injury of the lower legs or pelvis, use of walking aids, inability to give consent, pregnancy, and age under 18 years.

The healthy participants of both sexes (none of them identified as diverse) were fitted with OpenGO insoles (Moticon GmbH, Munich, Germany) matching their shoe size that were individually calibrated. Forces were recorded from both feet with one insole each. Measurements were conducted in the record mode of the device with a recording frequency of 100 Hz. Each insole is fitted with 16 pressure sensors. Raw data were exported for further analyses. The participants walked on a level treadmill at 4 km/h (Mercury, HP Cosmos, Nussdorf-Traunstein, Germany) for 1 minute, while insole data were collected. The participants were asked to walk for 1 minute straight, and recording was only commenced when the walking was already in progress to avoid bias by including altered steps upon gait initiation. Age was calculated from the date of birth. Body weight was measured with a Beurer MS 50 scale. Body height was measured with a Seca 206 roll-up measuring tape with wall attachment (Seca, Hamburg, Germany). Handgrip strength was measured with a hand dynamometer (Kern MAP 130K1, Kern, Balingen, Germany) by asking the participant while standing to hold the device in the dominant hand with the elbow extended and the arm hanging down (Ireland et al., 2014; Xu et al., 2021; Chen et al., 2022). Participants were asked to perform three maximal contractions with breaks, and the highest of the three values was chosen for statistical analysis. Handgrip strength is reported in kg. Of note, handgrip strength measured while standing with the elbow fully extended is greater than that measured while sitting (Xu et al., 2021).

### 2.2 Data management

The data obtained by the 16 force sensors in the insole devices were aggregated by the inbuilt data processing units and exported as described previously (Braun et al., 2015; Stöggl and Martiner, 2017). In detail, the pressure readings of the individual force sensors present in the insole device yield a weighted sum as the total vertical ground reaction force reading. Every summand is weighted by its sensor area (to compute the force) and a respective scaling factor accounting for the sensor’s surrounding area, as well as gaps between sensors, which also depend on the insole size. This process is conducted by the Moticon software as an automated processing step before the file export takes place. The raw data acquired by the devices were transferred to desktop computers and converted into csv-formatted ASCII-files. The resulting files were then loaded into a custom-developed data platform for further processing and parameter calculation.

### 2.3 Data processing

As a first processing step, stance phases of the gait cycles were identified and extracted from the time-series data (step detection). For this, any activity with consecutive force readings above 30N was considered. We applied a tolerance of up to three missing values due to possible recording device faults and discarded any activity with a duration of less than 300 ms or more than 2000 ms. To allow for inter-subject comparability of the derived gait parameters, normalization is required. Since the parameters presented in this publication are based on total force (force extrema and averages), as well as force over time (slope -based parameters), this applies to both the force and time axes. Force readings were transformed from Newton to proportion of body weight of the respective subject. It should be noted that since ground reaction force was measured instead of weight, due to acceleration, this value regularly exceeds the body weight for peak load bearing instances. Normalizing the time axis requires a more complex approach, as lack of a fixed cadence results in varying step activity lengths and thus amounts of true measurements for each step. Hence, a natural cubic spline interpolation was conducted on the original raw data. Based on the resulting curve for each stance phase, 100 equidistant samples were taken, yielding one (interpolated) force measurement point for every 1% of overall stance phase length.

When compared with other conventional gait measuring tools, such as sensor-equipped treadmills or ground-mounted force plates, insole sensors feature a lower recording frequency and higher sensor noise. Similar to the different gait phases, further parameters are usually based on or derived from the characteristic local extrema (first and second force peak, local minimum in between force peaks, which are also considered parameters themselves); sensor jitter can cause the issue of having multiple ambiguous candidates for those extrema. To remedy this problem, a Gaussian filter was applied to the original raw data while repeating the normalization process. This filtering strategy with the corresponding parameters (Sigma = 3, kernel size 7) prioritized the elimination of extrema ambiguity at the expense of signal precision, which can result in overcorrection in areas where a higher signal volatility is to be naturally expected (e.g., at the start and end of the stance phase). Therefore, in order to avoid losing high-frequency detail, the filtered and normalized curve was only used to determine unambiguous time-axis positions (indices) for the extremum candidates; those indices were then re-applied to the non-filtered, normalized data to yield the corresponding ground reaction force measurement closer to the original raw data. For cases in which using the filtered data still yielded inconclusive extremum candidates, additional detection strategies were applied in the following order: (a) Time plausibility: Extremum candidates occurring within the first or last 10 indices (first/last 10% of overall time span) are eliminated. (b) Max/Min-pool filtering: Should multiple extremum candidates occur within a pool size of five indices (equals to 5% of overall time span), choose candidate with highest/lowest force value. (c) Monotony-check: In case of multiple extremum candidates remaining, eliminate those candidates from which the curve does not display a strict monotonous decrease/increase in both directions within five indices each. (d) Monotony grace: In case monotony-check has eliminated too many candidates (less than two maximum candidates or less than one minimum candidate remaining), reinstate eliminated candidates in descending order of their highest achieved monotony distance until the target number of candidates is reached.

Every stance activity with an irregular amount of unambiguous extremum candidates remaining after the application of those strategies in the context of step detection was considered a non-step event, and thus removed from the dataset. In total, an average of 96.51 stance phase curves were extracted per participant, out of which an average of 7.35 had the additional extremum elimination strategies applied, as outlined above. An average of 14.86 events per participant had to be excluded over the entire experiment.

### 2.4 Parameters of the stance phase curve

**FIGURE 1:**
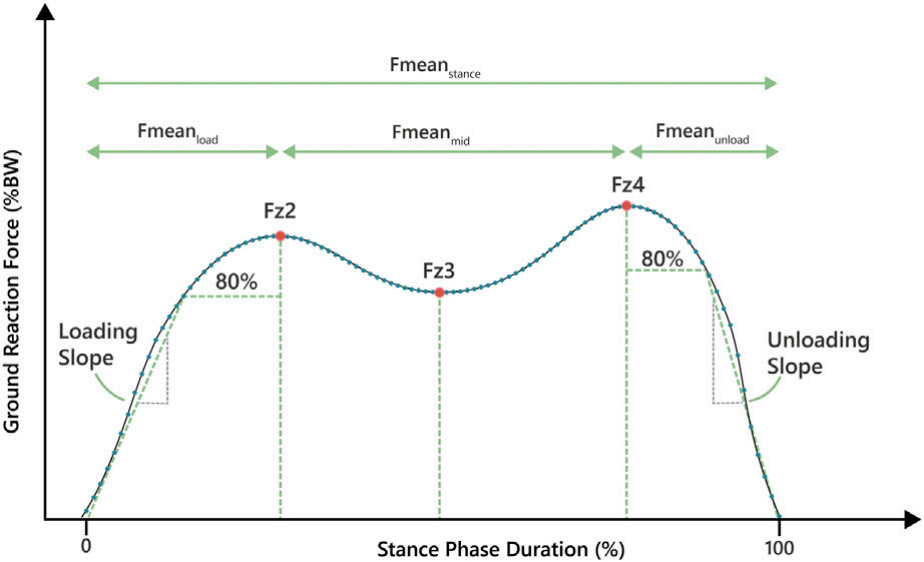
The plantar pressure gait curve with the determined parameters.

Figure 1 shows the analyzed parameters of the stance phase curve, which we computed for each step after applying the pre-processing procedure as described (Larsen et al., 2008; Stöggl and Martiner, 2017). In detail, the underlying curves for parameter extraction were not filtered, but are instead a product of the normalization process described in 2.3. Since the time-normalization in most cases produces more interpolated data points than the raw data provides, this can cause a smoothing effect similar to filtering. All curves were time-normalized to yield 100 force data points. The average was calculated by the mean of every data point at the same time index, respectively (0–99), which again produces a curve with 100 averaged force data points. All stance phase curves were extracted for each participant across the minute of walking. Mean forces were acquired using the arithmetic mean of all force values in a defined section given as proportions of body weight. Descriptions of all parameters are shown in Table 1.

**TABLE 1:** Parameter definitions and units.

Parameter name	Definition	Unit
Fmean_stance_	Mean force over the entire stance phase	% body weight
Fmean_load_	Mean force between start of loading-phase and Fz2	% body weight
Fmean_mid_	Mean force between Fz2 and Fz4	% body weight
Fmean_unload_	Mean force between Fz4 and the end of the unloading-phase	% body weight
Fz2	First force maximum marking the end of the loading-phase	% body weight
Fz3	Force minimum during the mid-phase between Fz2 and Fz4	% body weight
Fz4	Second force maximum marking the end of mid-phase and beginning of unloading-phase	% body weight
Loading slope	Slope of the line between the start of the loading-phase and the first force reading equal or higher than 80% of Fz2	% body weight/% stance phase duration
Unloading slope	Slope of the line between the first force reading in the unloading-phase below 80% of Fz4 and the end of the stance phase event	% body weight/% stance phase duration

### 2.5 Statistical analyses

All statistical tests were executed with IBM SPSS Statistics version 29 (IBM SPSS Statistics, Armonk, NY, United States). Normal distribution of data was tested by the Kolmogorov-Smirnov and Shapiro-Wilk tests. Significance was defined as *p* < 0.05. Multiple linear regression analyses were conducted with forced entry for each of the nine parameters of the stance phase trajectory separate (Figure 1; Table 1) as the dependent variable. Forced entry was chosen, as the number of independent variables is low and all variables have an explainable influence (Kucuk et al., 2016). The relationships of each of these parameters with age, weight, BMI, body height and handgrip strength as independent variables were explored. As body height, body weight and BMI intercorrelate, they could not be entered in the same model. Instead, two separate models were run, the main one including body weight and height, and a second one with BMI instead of body weight and height, but otherwise identical. Data visualization was conducted using the Matplotlib in Python. Due to lack of comparable data in the literature, the authors could not run an *a priori* sample size calculation. The sample size of 37 was an estimate based on what is common in the field, and taking into account the aim to measure a very diverse group of volunteers.

## 3 Results

**FIGURE 2:**
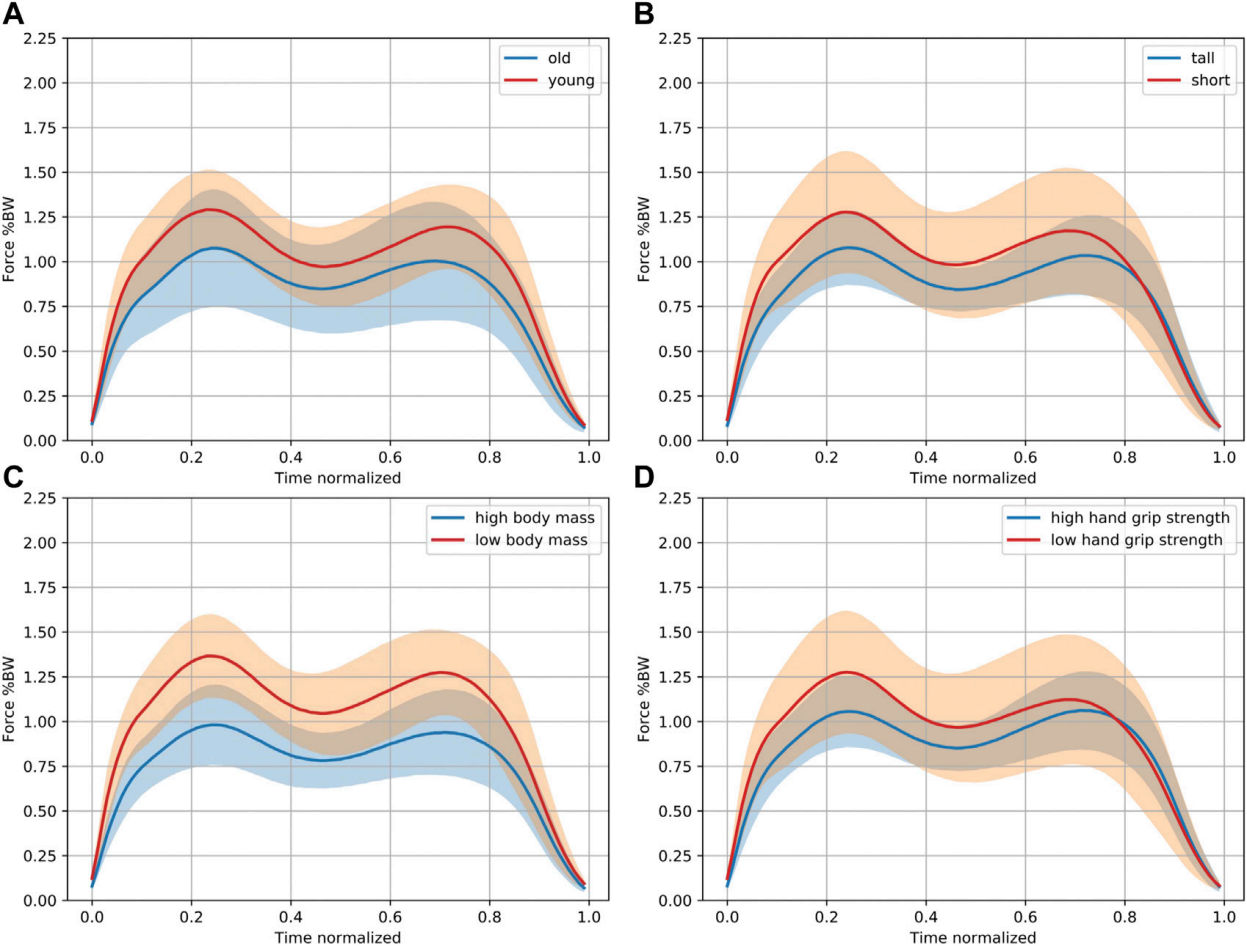
Characteristic differences in the stance phase curve. This figure is independent of the multiple regression analysis purely for illustration purposes. Pooled data of 18 out of 37 participants in each group are shown comparing the 18 **(A)** oldest and youngest participants, **(B)** tallest and smallest participants, **(C)** with the highest and lowest body mass, and **(D)** those with the highest and lowest handgrip strength. The solid lines indicate the mean. The 95% confidence intervals are shown in orange and blue, while the overlap shows up in brown. Higher normalized force values (in % body weight) can be observed in the younger and shorter participants with a lower body mass and handgrip strength.

A total of 37 participants (18 women and 19 men) with an average age of 43.65 ± 17.59 years were included in the study (for participant characteristics see Table 2). All models were significant, which means all models could be used. Table 3 shows the adjusted *R* ^2^ values and non-standardized and standardized (Beta) regression coefficients for all parameters. Figure 2 serves to illustrate differences in the trajectory of the insole-derived stance phase curve for younger and older, taller and shorter, heavier and lighter people, as well as those with a lower and higher handgrip strength.

**TABLE 2:** Participant characteristics.

	Total	Women	Men
N	37	18	19
Mean age [years] ± SD (range)	43.65 ± 17.59 (18–87)	38.35 ± 15.28 (23–65)	48.57 ± 18.49 (18–87)
Mean height [cm] ± SD (range)	173.70 ± 11.22 (157–203)	165.47 ± 6.03 (157–174)	181.23 ± 8.83 (163–103)
Mean weight [kg] ± SD (range)	79.81 ± 27.85 (43.9–170.8)	63.01 ± 13.42 (43.9–78.9)	96.67 ± 32.71 (63.4–170.8)
Mean BMI [kg/m^2^] ± SD (range)	22.78 ± 7.04 (13.81–45.63)	19.03 ± 3.98 (13.81–27.66)	26.56 ± 8.49 (18.11–45.63)
Mean handgrip force [kg] ± SD (range)	35.41 ± 12.46 (19.6–68.7)	26.14 ± 4.60 (19.6–38.0)	43.30 ± 11.59 (21.0–68.7)

**TABLE 3:** Adjusted *R* ^2^ values of the main model that includes body weight and height, and of the model that includes BMI instead. In addition, non-standardized and standardized (Beta) coefficients of the computed parameters are shown, if significant, separated by a comma. The values shown for BMI are derived from the BMI model, the others from the main model. Units are either kg, cm, or years per percent body weight, or in case of slope kg, cm or years per percent body weight per percent stance phase duration. The non-standardized coefficients can be used to correct for age, height, weight, BMI and handgrip strength when analysing such data.

	Adjusted *R* ^2^ (main model)	Adjusted *R* ^2^ (BMI model)	Age [years]	Body height [cm]	Body weight [kg]	BMI [kg/m^2^]	Handgrip strength [kg]
Fmean_stance_ [% body weight]	0.400	0.365			−0.007, −0.882	−0.022, −0.761	0.120, 0.696
Fmean_load_ [% body weight]	0.460	0.391		−0.008, −0.413	−0.005, −0.715	−0.018, −0.595	
Fmean_mid_ [% body weight]	0.285	0.267			−0.007, −0.818	−0.026, −0.716	0.130, 0.652
Fmean_unload_ [% body weight]	0.335	0.338			−0.006, −0.946	−0.019, −0.831	0.010, 0.764
Fz2 [% body weight]	0.376	0.350			−0.008, −0.748	−0.028, −0.646	
Fz3 [% body weight]	0.199	0.182			−0.006, −0.734	−0.020, −0.646	0.012, 0.672
Fz4 [% body weight]	0.282	0.278			−0.010, −0.899	−0.035, −0.790	0.019, 0.763
Loading slope [% body weight/% stance phase duration]	0.292	0.177	−0.067, −0.394	−0.145, −0.543			
Unloading slope [% body weight/% stance phase duration]	0.264	0.255			0.083, 0.835	0.283, 0.723	−0.150, −0.677

### 3.1 Age

The only parameter of the stance phase curve that showed a significant negative correlation with age was the mean loading slope (*p* = 0.014). Younger participants had higher loading slope values, indicating a steeper increase. All other analyzed parameters were independent of age.

### 3.2 Body height

The body height correlated negatively with Fmean_load_ (*p* = 0.046) and the loading slope (*p* = 0.023). All other analyzed parameters were independent of body height.

### 3.3 Body weight and BMI

The body weight correlated with all the analyzed parameters of the stance phase, except the loading slope (Fmean_stance_: *p* < 0.001, Fmean_load_: *p* = 0.002, Fmean_mid_: *p* = 0.002, Fmean_unload_: *p* < 0.001, Fz2: *p* = 0.002, Fz3: *p* = 0.007, Fz4: *p* < 0.001, unloading slope: *p* = 0.002). Identical to the body weight, the BMI (separate model) also correlated with all parameters, except the loading slope (Fmean_stance_: *p* < 0.001, Fmean_load_: *p* = 0.005, Fmean_mid_: *p* = 0.002, Fmean_unload_: *p* = 0.002, Fz2: *p* = 0.003, Fz3: *p* = 0.008, Fz4: *p* < 0.001, unloading slope: *p* = 0.002).

### 3.4 Handgrip strength

The handgrip strength correlated positively with the parameters of the mid and unloading phase, Fmean_stance_ (*p* = 0.015), Fmean_mid_ (*p* = 0.036), Fmean_unload_ (*p* = 0.012), Fz3 (*p* = 0.041), Fz4 (*p* = 0.015) and the unloading slope (*p* = 0.032). There was no correlation with Fmean_load_, Fz2 and the loading slope, which are the parameters of the early stance phase.

### 3.5 Variability

The adjusted *R* ^2^ values shown in Table 3 indicate that only up to 46% of the variability in the analyzed parameters can be explained by age, body weight, height, BMI and hand grip strength. Thus, further factors must affect the trajectory of the gait cycle curve, that have not been considered in the present analysis.

## 4 Discussion

In summary, the present study demonstrated influences of age, body height, body weight, body mass index and handgrip force with parameters derived from the trajectory of the stance phase curve in healthy participants. However, only up to 46% of the variability in the analyzed parameters can be explained by age, body weight, height, BMI and hand grip strength. Thus, further factors affect the trajectory of the gait cycle curve, that have not been considered in the present analysis. When analyzing insole data, it might be useful to use the identified factors as input for machine-learning algorithms, or to correct for them by using the regression coefficients presented in this paper.

Pressure insoles are increasingly used to study gait in patients, as well as for lifestyle and health monitoring (Braun et al., 2017; Subramaniam et al., 2022). In addition to continuous measurements with insoles in patients with injuries, insoles are used in neurological patients for home-based treatment monitoring and as a rehabilitation tool for neuro-impaired gait, such as in Parkinson’s disease (Sica et al., 2021; Das et al., 2022). Such long-term monitoring, especially if combined with further sensors, may produce large amounts of data that require automated analyses. Among the possibilities is the use of machine-learning algorithms trained with annotated data for pattern recognition of which activities a person is performing or has been able to perform (Harris et al., 2022). Such algorithms could be trained to recognize not only level walking and running, but also activities such as climbing stairs, cycling, driving a car, riding a train or bus, indicate falls and events with excessive loads, and quantify the overall time a person has been active. In addition, prediction algorithms could be implemented for falls and diseases. Machine-learning algorithms are already in use for many applications in gait analyses, including spatiotemporal human gait recognition (Harris et al., 2022; Konz et al., 2022). Such analyses in real time open up the possibility to deliver patient feedback, including warning of excessive forces and movements, as well as to remind patients that it is time to get up and exercise (Zheng and Chen, 2017). The inherent limitations in computing power of small wearable devices are increasingly targeted by both algorithmic optimization techniques in machine-learning like reservoir computing, network pruning, dimensionality reduction and so on, as well as hardware innovations. Together, these advances will ultimately allow real-time feedback based on data from various sources combined in the near future (Chiasson-Poirier et al., 2022; Zhang et al., 2022). Alternatively, extracting decision making systems (symbolic artificial intelligence), such as threshold-based methods, might offer an immediate route to real-time feedback.

As shown in the present study, individual characteristics of gait should be taken into account when analyzing continuous gait data in the field and daily life, and especially when deriving advice and warnings or alarms from these data. The present study revealed that the loading slope decreased with increasing age. This finding is novel, but in line with previous studies that showed a more cautious gait (more focus on gait combined with a slower walking speed) as well as age-related reductions of the preferred walking speed, and cadence (Herssens et al., 2018; Niederer et al., 2021). The more cautious gait in the present study is reflected in the slower loading of the foot, while the force maxima did not change with age. Larsen et al. (2008) showed that elderly compared to young participants had elevated muscular coactivation along with greater electromyographic activation when walking stairs, which illustrated the more cautious gait. Interestingly, in a different study, in participants 70 years and older, the center of mass push-off power was shown to be significantly decreased (Sloot et al., 2021). This effect did not show up in the present study, likely as the average participant age was relatively low. The finding, however, matches the increasing decline in performance with age that accelerates from the age of around 70 years onward (Ganse et al., 2020; Ganse et al., 2021). It should thus be assumed that the force maxima of the plantar pressure stance phase curve might decrease in the oldest-old, and particularly the second maximum that reflects the push-off force.

The body height affected Fmean_load_ and the loading slope, and thereby delivered a characteristic change in the curve trajectory. This finding is not surprising, as devices are usually calibrated to body weight, but not to body height. Known changes in gait characteristics in taller participants include lower cadence due to longer steps, lower ankle velocity due to fewer steps, and increased stride time and stride length (Mikos et al., 2018). Taller people with longer legs often prefer higher walking speeds than smaller people. Walking speed has been shown to impact spatio-temporal gait parameters, and the perception of what the most comfortable walking speed is certainly varies among individuals (Andriacchi et al., 1977; Kirtley et al., 1985). The walking speed of 4 km/h, however, that was used in the present study, is usually in the range of what healthy participants consider comfortable. In patients with walking impairments, however, 4 km/h might be too fast, and slower speeds should be selected when studying gait (Linder et al., 2022; Zhu et al., 2022).

The body weight and BMI both had an influence on all the analyzed parameters of the stance phase curve, except the loading slope. The latter is an interesting detail, since age only affected the loading slope, which allows to determine the influence of weight/BMI independent from the effect of age.

The handgrip strength affected the mean force values of the later stance phase curve, the minimum and the push-off force (second maximum), as well as the unloading slope. There was, however, no effect on the average force of the initial phase, the first maximum and the loading slope. This very clear picture shows that the handgrip strength correlates only with the second half of the stance phase curve, while it leaves the first half unaffected. The underlying cause is likely the muscle force available for push-off, that is usually stronger in individuals who also have more handgrip strength. The hip abductors and adductors contribute the most muscle power in adult normal walking and are thus the muscles that would need to be studied more closely in future studies that deal with correlations of muscle strength and gait (Bogey and Barnes, 2017).

In future studies, also further parameters should be assessed that may affect the stance phase curve to enable for advanced analyses in the daily life. These may include the shoe and surface type, as well as a variety of characteristic activities. In addition, children and people aged 70 years and older should be studied. It is currently unknown how different injury types and degenerative musculoskeletal conditions affect the gait cycle curve, such as bone fractures, ligament injuries or arthritis of the legs. In case characteristic differences can be found among injury types, analyses of the stance phase curve may even have diagnostic and potentially even predictive value.

The main limitation of the study is that the effect of walking speed was not considered, as only one pre-defined walking speed was used for the measurements. Another limitation is that the data were recorded on a treadmill that is known to differ from walking on a ramp or on normal ground (Strutzenberger et al., 2022). The setup and walking speed were chosen to get the best standardization possible. In addition, differences in the pressure distribution under the feed *via* the 16 sensors were not analyzed per sensor in the present study and could be assessed in the future. Of note, the parameters analyzed in the present study can only be used when a regular gait curve is present. If this is not the case, other methods need to be applied, i.e., machine-learning or the analysis of other parameters, including possibly slopes and averages.

## 5 Conclusion

Age, body height, body weight, body mass index and handgrip force affect the trajectory of the stance phase curve in characteristic ways, but only explain up to 46% of the variability. When analyzing insole and ground-reaction-force data, it might be useful to correct for such factors, especially in automated data analyses *via* wearables, when the data are analyzed to give warnings and deliver live feedback. Smart healthcare applications that use insole data could help to improve patient health and facilitate treatment after injuries.

## Data Availability

The original contributions presented in the study are included in the article/Supplementary Material, further inquiries can be directed to the corresponding author.
